# Examining the subjective fairness of at-home and online tests: Taking *Duolingo English Test* as an example

**DOI:** 10.1371/journal.pone.0291629

**Published:** 2023-09-19

**Authors:** Don Yao

**Affiliations:** Department of English, Faculty of Humanities and Foerign Languages, China Jiliang University, Hangzhou, China; Chulalongkorn University, THAILAND

## Abstract

The Duolingo English Test, a language proficiency test offered online, is now getting prevalent worldwide. A recap of existing literature denotes that there is an insufficient examination of the DET, particularly on its issues of fairness. Besides, empirical test fairness research had mainly focused on the objective aspect but may have overlooked the importance of its subjective aspect. Additionally, compared with in-person tests, the fairness research of at-home tests lags far behind. Therefore, the current study investigated the DET fairness from test takers’ perspectives. A DET Fairness Questionnaire based on (Kunnan’s AJ, 2004) Test Fairness Framework (comprising validity, absence of bias, access, administration, and social consequences) was developed. Data were collected from 1,012 Chinese university students and processed through descriptive and factor analyses. The descriptive analyses revealed that test takers perceived the DET to be fair overall. Specifically, they perceived that they had equal access to the test, but the test was invalid; the factor analyses showed that test takers’ perceptions of DET fairness (especially perceived validity and access) had a significant effect on their test performance. Such findings suggest that the subjective test fairness as an essential component could not be neglected in appraising an assessment as it influences test takers’ performance, and DET developers may strive harder to enhance the validity of DET to provide a fairer testing environment for test takers.

## 1 Introduction

In recent years, an increasing number of Chinese students have pursued further studies abroad [1]. To enroll in English-medium universities, students are required to demonstrate their sound English written and spoken skills [2]. In most cases, students’ English language proficiency is represented by the grades they get in standardized English proficiency tests, e.g., TOEFL and IELTS. Chinese students, therefore, have strong desires and strive hard to pass various English language tests [3]. However, due to the Corona Virus Disease-19 (COVID-19) pandemic, in-person administrations of these tests were disrupted under the worst circumstances. Subsequently, the Duolingo English Test (DET), a language proficiency test offered at home and online, became prevalent worldwide [4, 5].

The DET is an English proficiency test assessing non-native English test takers’ integrated abilities, i.e., literacy, conversation, comprehension, and production. It is displayed on the Duolingo official website (https://englishtest.duolingo.com) that the test is: (1) convenient: test takers can take the test online anytime and anywhere-no traveling to a test center or appointment needed; (2) fast: test takers will get results in two days of completing the test, and share it with anymore, immediately; (3) and affordable: the test charges only $49 while most other certification tests cost over $200. Also, over 3,000 educational intuitions have now acknowledged this computer-adaptive test for university admissions or as a measure of applicants’ English proficiency. Apart from university admissions, the test could also be used for middle or high school admissions and job applications or promotions.

However, the test has received much skepticism in terms of its fairness (e.g., security, accessibility, item authenticity, and possible negative impact on teaching on learning) since its launch [4, 5]. For instance, Wagner [4] contends that though the DET has a rigorous remote invigilation system for test administration, it is hard to avoid the potential for cheating. He also doubts whether the DET could improve their test takers’ English language proficiency in real-world contexts. Besides, Isbell and Kremmel [5] note that at-home tests (e.g., DET) may leave a problem for test takers who are not able to afford a personal computer or access fast and stable internet connections. Finally, they all maintain that the DET includes many low-context and discrete items which may fail to measure test takers’ English proficiency in academic contexts, though it is widely used for university admissions purposes. In the arena of language assessment, it is claimed that an assessment ought to be fair to all test takers [6]. The DET is getting prevalent worldwide, but its fairness issues have not been extensively researched. Given the high-stakes and large-scale nature, it is necessary to examine the fairness issues of the DET.

A review of empirical literature suggests that prior DET research is scarce. Only a total of nine DET research reports have been published on the Duolingo official website mainly examining its validity issues (e.g., [7–9]), and three test reviews reviewing the DET from its test quality, technology, and security [4, 5]. Among research reports, researchers have gradually sensed the significance of DET fairness, but they have only drawn upon the aspects such as test security and absence of bias. Regarding test fairness, empirical studies motivated by fairness theories are not many. Also, previous research has focused on objective test fairness (i.e., the examination of the psychometric qualities of the test; [10]), but the research on subjective fairness “that thoroughly examines the perceptions of test takers concerning fairness” is scarce [11] (p. 1). Nevertheless, Wallace [12] believes that subjective fairness is of great importance as stakeholders (e.g., teachers and students) are affected by an assessment the most, indicating that their perceptions of test fairness are also significant [11].

To bridge the gaps, the present study sought to explore the fairness of the DET from test takers’ perspectives. Given that Chinese test takers hold the largest testing population of DET globally, their perceptions of the test may merit more scholarly attraction [13]. The study was quantitative-oriented utilizing a questionnaire survey design to generalize the research findings. Descriptive analysis was first conducted to obtain a general understanding of test takers’ DET fairness perceptions. Exploratory factor analysis (EFA) and confirmative factor analysis (CFA) were employed to process questionnaire data. Structural equation modeling (SEM) was adopted to model the complicated quantitative relationships between test takers’ perceptions of DET fairness and test performance. The results from the study may bring about profound practical implications to the arena of language assessment.

## 2 Literature review

### 2.1 DET research reports and DET test reviews

As displayed in Table 1, seven out of nine studies have delved into DET’s criterion-related validity. Most DET researchers devoted themselves to aligning the DET scores to other standardized tests’ scores such as TOEFL [14], IELTS [15], or both [16–18]. Another focus the DET researchers have paid attention to is the score reliability. A total of five out of nine reports investigated the reliability of DET scores, e.g., test-retest reliability or both the test-retest reliability and internal consistency [17–19]. Except for scrutinizing the criterion-related validity and score reliability of the DET, previous reports have also explored other DET aspects: e.g., Bézy and Settles [15] examined whether the DET scales could be aligned to CEFR levels; Maris [8] investigated the construct validity of the DET, and whether there was a statistical bias toward different groups of test takers; and Settles and LaFlair [17] examined the item bank security.

**Table 1:** Empirical DET research reports.

Author/Year	Focus/Foci
Ye [14]	Criterion validity (TOEFL), test-retest reliability
Bézy and Settles [15]	Criterion validity (IELTS), alignment to CEFR
Ishikawa et al. [7]	Criterion validity (TOEFL, on-campus faculty assessments)
Settles [19]	Internal consistency, test-retest reliability
Brenzel and Settles [16]	Criterion validity (TOEFL, IELTS)
Maris [8]	Construct validity, absence of bias
Settles and LaFlair [17]	Criterion validity (TOEFL, IELTS), internal consistency, test-retest reliability, security
LaFlair [9]	Sub-scores’ criterion validity (TOEFL, IELTS), Sub-skill scores’ internal consistency, test-retest reliability
LaFlair and Settles [18]	Criterion validity (TOEFL, IELTS), internal consistency, test-retest reliability

Results from these reports are that: (1) the DET scores are moderately to largely correlated to the TOEFL and IELTS scores, (2) the DET scores are reliable; (3) the DET scales could be partially aligned to CEFR levels; and (4) the DET is in good shape in terms of its language abilities assessed; (5) there is no evidence of statistical bias toward different groups of test takers regarding item types or computers; and (6) the DET has a large item bank. These results are mostly related to the validity issues of the DET, and they reflect the positive side of the test.

However, Wagner [4] and Isbell and Kremmel [5] critically reviewed the DET, and the negative side of the DET has been emerging. Both reviews have listed several potential shortcomings of the DET (or at-home tests), especially in terms of its fairness issues (e.g., the possibility of cheating on the test, potential negative impact on teaching and learning, and affordability of personal laptop). Also, both reviews mention that the DET has little in common with the university contexts, and the score uses need further negotiation. Given the high-stakes and large-scale nature of the DET and the potential problems that the test may have, it is necessary to conduct a study to comprehensively examine the fairness of the test. The following section, therefore, reviews the concept of test fairness and related research.

### 2.2 Test fairness

Test fairness is considered an essential aspect in the evaluation of language assessments [10, 12, 20, 21]. An early discussion of fairness in assessment was provided in the *Code of Fair Testing Practices in Education* (hereafter Code), a guide for professionals (i.e., test developers and test users) in fulfilling their obligations to offer and use assessments that are fair to all test takers in education. It defines a fair assessment as one that avoids content or language, race, gender, and ethnic bias, ensures construct relevance, and provides accommodations with disability [22]. Later, the concept of test fairness was discussed in the Standards for Educational and Psychological Testing (Standards from now on), written for professionals (e.g., test developers, test users, sponsors, publishers, and employers) to address various standards of test development and use in education, psychology, and employment. It conceives test fairness as absence of bias, equitable treatment of all test takers in the testing process, and equity in opportunity-to-learn (OTL) the material in an achievement assessment [6, 23]. It could be found that the definition of test fairness in the Code is in accordance with that in the Standards, but the fairness statements in the Standards could be utilized in the broader scope of audience and purpose compared with those in the Code [24, 25].

**Fig 1:**
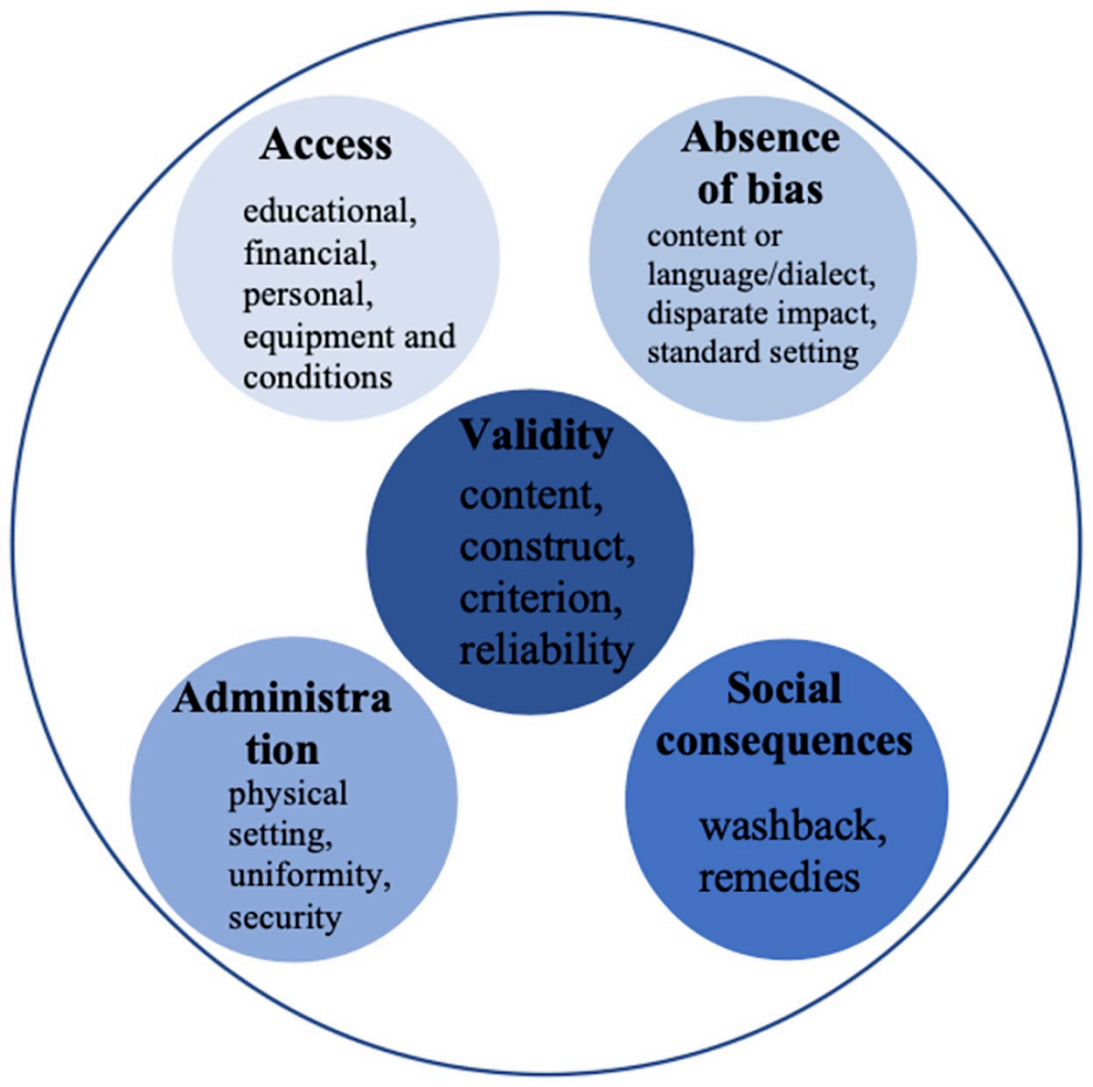
Kunnan’s [27] Test Fairness Framework.

Despite its importance in the theoretical literature, assessment fairness was not accounted for in the empirical literature, Kunnan [26] reviewed appropriate 100 validation studies in language assessment and reported that the themes addressed by researchers are not particularly related to fairness. To better understand test fairness, it is conceived as a three-notion concept on the grounds of the Code and the Standards: validity, access, and justice [20]. It is these qualities that form the backbone of the Test Fairness Framework (TFF) that Kunnan [27] proposed in 2004. The TFF has five major qualities: validity (content representation and relevance, construct or theory-based validity, criterion-based validity, and reliability), absence of bias (offensive content or language, language, disparate impact, and standard-setting), access (educational, financial, geographical, personal, equipment, and equipment and conditions), administration (physical setting and uniformity and security), and social consequences (washback and remedies; [27]; see Fig 1). This framework “views test fairness in terms of the whole system of a testing practice not just the test itself” ([27], p. 2; [28], p. 235). That said, multiple facets of test fairness might be implicated: test uses (intended or unintended uses), stakeholders (test takers, test users, teachers, and employers), and test development (test design, development, administration, and use).

### 2.3 Empirical research related to the TFF

Kunnan’s [27] TFF, prioritizing the role of test fairness, has broadened the span of fairness. The framework has been adopted in the research that investigates the fairness of large-scale assessments (e.g., [29, 30]) and small-scale assessments (e.g., [10, 12]). For large-scale assessments, Hamid et al. [29] examined the fairness and justice of the IELTS and found a large proportion of participants believed that the test was not an accurate measure of their language abilities, and they were not satisfied with the IELTS justness in that they were not sure whether the test was a vehicle for increasing income or its suitableness for immigration purposes. Moghadam and Nasirzadeh [30] investigated the fairness of a locally developed English proficiency test and found that the test was acceptably fair from the perspectives of access, administration, and social consequences. But the validity of the test should be enhanced; and one item was flagged as gender DIF which needed further revision. As for small-scale assessments, Wallace and Qin [10], in line with Kunnan’s [21, 27] view of test fairness and justice, divided fairness into four dimensions (i.e., distributive, procedural, interactional, and informational fairness) and probed test takers’ perceptions of classroom-based assessment fairness and concluded that test takers felt the test procedures and their communications with teachers were both fair; nevertheless, they were conservative about score interpretations. Later in 2021, Wallace and Qin [10] conducted a replication study in another Chinese city and outcomes were almost the same as the previous research’s.

In brief, Kunnan’s [27] TFF has been attracting certain scholarly attention, but there lacks a study comprehensively investigating the fairness issues of an assessment with the help of this framework. Besides, previous research has examined the fairness issues of both large-scale (e.g., IELTS and CET-4) and small-scale assessments (e.g., classroom-based tests). As a high-stakes and large-scale assessment, the fairness issues of the DET have not been systematically researched yet. Furthermore, Wallace [12] argues that most language tests are regarded as fair only based on the psychometric examinations of the test or other test-internal technical qualities, and Choi [11] also notes that “there is no research that thoroughly examines the perceptions of test takers concerning fairness” (p. 1). However, being affected by an assessment the most, stakeholders’ (e.g., test takers’) perceptions of test fairness are also of great importance [11, 12]. The following sub-section, then, introduces Wallace [12] objective and subjective test fairness.

### 2.4 Wallace’s [12] objective and subjective test fairness

Drawing upon the TFF, Wallace [12] further separated test fairness into objective fairness and subjective fairness. Objective fairness refers to the examination of the psychometric qualities of the test [10]. Specifically, an assessment is considered objectively fair if: (1) it is statistically valid, reliable, and absent of bias; (2) it provides equal access and OTL to all test takers; (3) it is administered equally; and (4) it is beneficial to society. Subjective fairness refers to the extent to which the stakeholders (e.g., students or teachers) perceive that the test is valid, and absent of bias; has equal access and administration provided and beneficial social consequences. That said, it is “determined by the perceptions of the students and teachers” (p. 493). It is argued that subjective fairness also attaches great importance as it considers stakeholders’ (who are directly affected by an assessment the most) views toward the fairness of an assessment [11, 12].

### 2.5 Conceptual model and research questions

**Fig 2:**
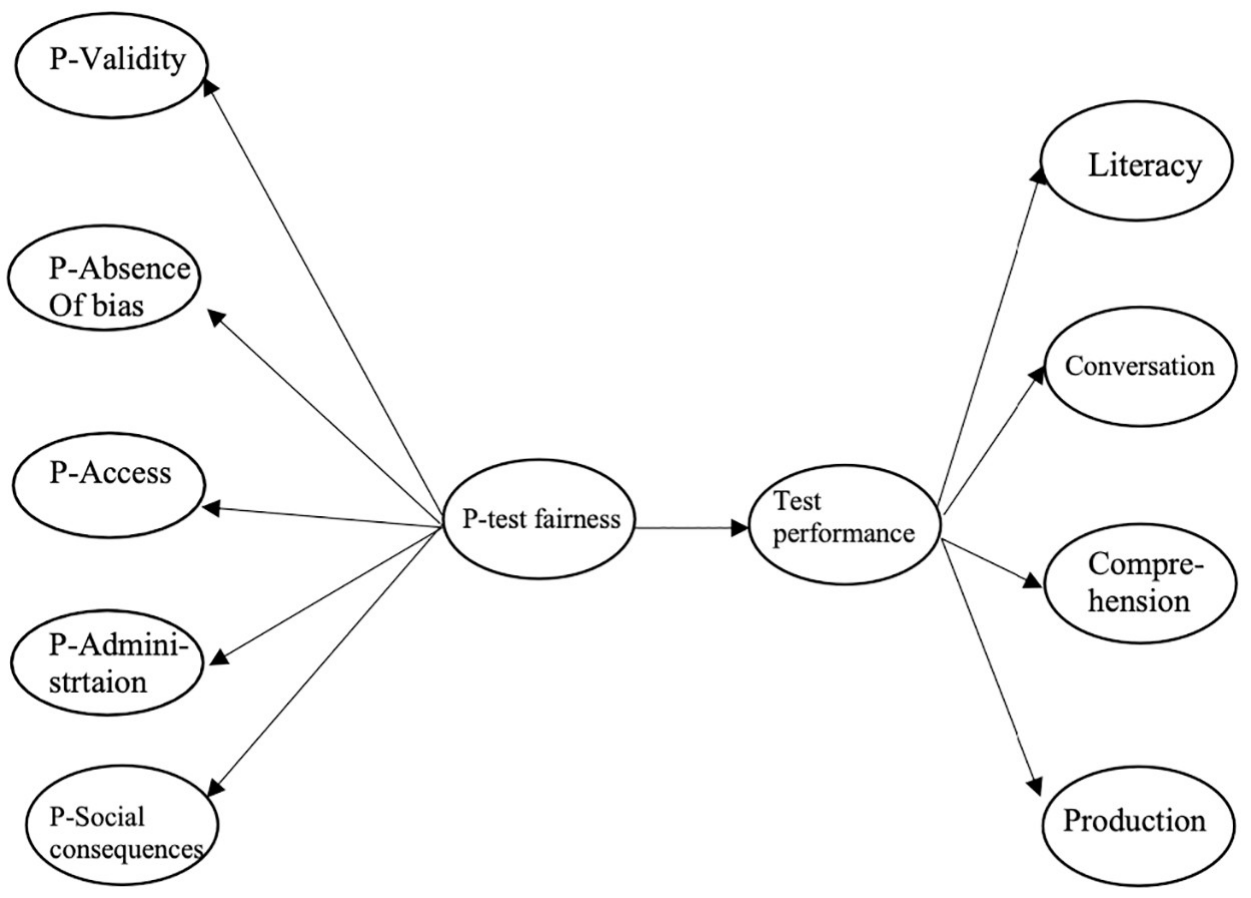
Conceptual model. Note. P = Perceived.

In accordance with Wallace’s [12] view of subjective fairness and with the help of Kunnan’s [27] TFF, the current study intends to extensively understand test takers’ perceptions of DET fairness. A conceptual model, therefore, has been proposed in the current study (see Fig 2). The model incorporates two vital elements: perceived test fairness (including perceived validity, perceived absence of bias, perceived access, perceived administration, and perceived consequences) and test performance. Also, two research questions have been articulated:

- **RQ 1: How do test takers perceive the fairness (i.e**., **validity, absence of bias, access, administration, and social consequences) of the DET?**
- **RQ 2: How does perceived DET fairness affect test performance?**

## 3 Methods

### 3.1 Participants

A total of 1,098 (M = 547; F = 551) participants were recruited in the current study. After data cleaning, 86 responses were removed for further analyses. The rest 1,012 participants (M = 498, 49.21%; F = 514, 50.79%) are aged from 21 to 24 (21 = 24.41%, 22 = 26.38%, 23 = 23.22%, and 24 = 25.99%). They are all Chinese, and their first language is Chinese Mandarin or Cantonese, and their second language is English. They all have been learning English for more than 12 years. They prepared for the DET for an average of six weeks, and they took the test averagely twice. Finally, their English proficiency represented by DET scores varies. Most participants scored DET from 90 to 140 (90 to 115 = 41.10%; 120 to 140 = 46.14%), a few participants scored 85 and below (10.00%), and few participants scored 145 and above (2.76%). In light of the score alignment to CEFR levels from the DET official website, it reveals that most DET test takers’ English proficiency is at CEFR B2 and C1 levels.

### 3.2 Instrument

A DET Fairness Questionnaire was developed based on Kunnan’s [27] TFF. The questionnaire was piloted among 109 participants first, and the overall reliability value was .739. Two items were found slightly problematic and, hence, revised in the finalized version. The whole questionnaire is composed of two sections (see S1 Appendix). Section A asks for participants’ demographic information, e.g., age, gender, region, and DET scores; and Section B with 25 items is linked to participants’ perceived test fairness: perceived validity (items No. B1 to B5), perceived absence of bias (items No. B6 to B10), perceived access (items No. B11 to B15), perceived administration (items No. B16 to B20), and perceived social consequences (items No. B21 to B25). All items are constructed on a six-Likert scale, and participants are required to choose only one option (from 1 = strongly disagree to 6 = strongly agree) that fits them the most. Both English and Chinese versions are provided.

### 3.3 Data collection

The online survey tool Wenjuanxing (see https://www.wjx.cn/ for the layout) was used for questionnaire distribution. Wenjuanxing is a user-friendly research tool for online survey questionnaires widely used, especially in mainland China. It has more than 50 types of functions and is easy to design the questionnaire. Before the questionnaire delivery, face validity was verified.A Ph.D. candidate majoring in English linguistics helped check whether the tool functioned well. Also, she finished the questionnaire and reported the estimated time to fill out the questionnaire (around 10 minutes). Revisions were made based on her suggestions and comments. At the beginning of the questionnaire, introduction, purpose, procedure, confidentiality, and contacts and questions were explicitly stated. Participants were informed that the data were confidential and used only for academic purposes. Besides, written consent was obtained from all participants which was listed on the first page of the questionnaire. For Section A, participants were required to input their demographic information; for Section B, they had to choose the option (from 1 = strongly disagree to 6 = strongly agree) fitting them the most. There was no time limit for each participant, but all items were set as compulsory to answer to avoid the problem of missing values in further data analyses.

The participants were recruited through both snowball sampling and convenience sampling [31, 32]. For one thing, the researcher posted the questionnaire onto social media with a list of requirements (e.g., took the DET before, gender, years of learning English, and duration of test preparation). People who were eligible could fill out the questionnaire. For another, the researcher sought help from the data collection agency Wenjuanxing in Mainland China. The company asked for the whole questionnaire and the requirements that the researcher had, and they helped collect data in two weeks and sent back the researcher a dataset with all information required.

### 3.4 Data analysis

Data analysis was divided into descriptive analysis and factor analysis. The software SPSS v.26.0 and AMOS v.26.0 were used for different statistical analyses. The descriptive statistics were displayed to gain a general idea of test takers’ perceived DET fairness. Factor analysis was conducted to model the complicated quantitative relationships: EFA was first conducted at the item level to figure out whether items designed to measure the same underlying construct loaded together; CFA was then conducted at the sub-scale level to assess the reliability and internal validity of each measurement model; and SEM was applied to model the relationships between perceived DET fairness and test performance.

## 4 Results

- **RQ 1: How do test takers perceive the fairness (i.e**., **validity, absence of bias, access, administration, and social consequences) of the DET?**

The internal consistency of the finalized questionnaire was first computed. The result shows that the overall Cronbach’s Alpha coefficient is .807, which is higher than the pilot version’s (α = .739). This result suggests the questionnaire is reliable and could be used for further analyses.

### 4.1 Descriptive analysis

Table 2 enumerates the descriptive results of participants’ perceived DET test fairness. Both skewness and kurtosis are within -2 to +2, indicating the dataset is normally distributed, which could be used for further parametric statistical analysis. From the table, it could be found that participants perceive the DET to be fair overall (Mean = 3.535; S.D. = 1.075). They perceive that: (1) the items on the DET are not biased (Mean = 3.578; S.D. = 1.156); (2) they have equal access to the test (Mean = 3.927; S.D. = 1.034); (3) the conditions of administration are equal to them (Mean = 3.588; S.D. = 1.158); (4) the DET brings about positive social consequences (Mean = 3.721; S.D. = 1.153); but (5) the DET is not valid (Mean = 2.860; S.D. = 1.186).

**Table 2:** Descriptive results (n = 1,012).

Item No.	Mean	Std. Deviation	Skewness	Kurtosis
**Validity**	**2.860**	**1.186**	**.358**	**-1.206**
B1: construct	3.245	1.057	.398	.310
B2: content	2.632	1.033	.222	-.015
B3: criterion	2.476	1.012	.245	.167
B4: reliability	2.671	.823	.553	-.371
B5: reliability	3.278	.857	.104	-.970
**Absence of bias**	**3.578**	**1.156**	**.058**	**-1.018**
B6: language/dialect	3.508	1.123	.021	-1.373
B7: gender	3.623	1.087	.153	-.927
B8: religion	3.712	1.088	.073	-1.431
B9: age	3.532	1.021	-.838	.763
B10: standard setting	3.511	1.001	-.843	1.081
**Access**	**3.927**	**1.034**	**-.795**	**.532**
B11: opportunity to learn	3.877	1.021	-.738	.589
B12: affordability	4.125	1.123	-.821	.792
B13: location	3.879	1.047	.283	-.823
B14: accommodation	3.756	1.123	.354	-.781
B15: time	3.996	1.127	.280	-.804
**Administration**	**3.588**	**1.158**	**.291**	**-.853**
B16: physical condition	3.429	1.167	.300	-.811
B17: uniformity	3.816	1.076	-.051	-.971
B18: disability	3.624	1.087	-.123	-.943
B19: procedure	3.483	1.096	-.083	-.961
B20: security	3.587	1.123	-.033	-1.005
**Social consequences**	**3.721**	**1.153**	**-.140**	**-.800**
B21: remedy	3.575	1.034	-.389	-.594
B22: washback	3.967	.934	-.369	-.697
B23: washback	3.865	.927	-.083	-.872
B24: washback	3.517	1.036	-.033	-.998
B25: washback	3.684	1.068	-.140	-.870
**Overall**	**3.535**	**1.075**	**.998**	**-1.233**

Note. No. = Number; Std. Deviation = Standard Deviation. Brief introduction of each item was provided. For specific items, please refer to the S1 Appendix.

To be more specific, for the absence of bias quality, participants perceive that items on the DET are not biased in terms of gender (Mean = 3.623; S.D. = 1.087) and religious belief (Mean = 3.712; S.D. = 1.088). That said, they perceive that items on the DET do not favor male or female test takers, or test takers with different religious beliefs. For the access quality, participants perceive that the price of the DET is appropriate (Mean = 4.125; S.D. = 1.123), and choosing the test time themselves make taking the test easy (Mean = 3.996; S.D. = 1.127). For the administration quality, participants perceive that test takers should take different test forms to alleviate the possibility of cheating on the test (Mean = 3.816; S.D. = 1.076). For the quality of social consequences, participants perceive that their English proficiency is improved after taking the DET (Mean = 3.967; S.D. = .934), and the DET helps their English learning (Mean = 3.865; S.D. = .927). Nevertheless, for the validity quality, participants perceive that the content on the DET may not be appropriate to measure their language ability (Mean = 2.632; S.D. = 1.033), the DET score may not be able to compare to scores from other proficiency tests such as IELTS and TOEFL (Mean = 2.476; S.D. = 1.012).

- **RQ 2: How does perceived DET fairness affect test performance?**

### 4.2 Factor analysis

#### 4.2.1 EFA results

EFA was first conducted at the item level to determine whether items designed to measure the same underlying construct loaded together. A total of 337 participants’ questionnaire responses were randomly selected. The process is divided into five steps [33]. The first step is to check whether the dataset is suitable for factor analysis. The Kaiser-Meyer-Olkin (KMO) value of the perceived test fairness construct is .926, which is above the cut-off value of .60 [34]. Besides, Bartlett’s test of sphericity confirms the adequacy of the magnitude of the correlations by displaying a Chi-square value of 5433.300 (p< .001), which is significant. These two results reveal that the dataset is suitable for further analyses.

The second and third steps are to extract factors. The purpose of these steps is to classify items into different factors. In the second step, the factor extraction method needs to be confirmed. Given that the dataset is normally distributed, the Maximum Likelihood method was used [35–37]. After ensuring the extraction method, the item communality was checked. It is found that all values are above .30 [38], representing that all items are suitable for further analyses.

**Fig 3:**
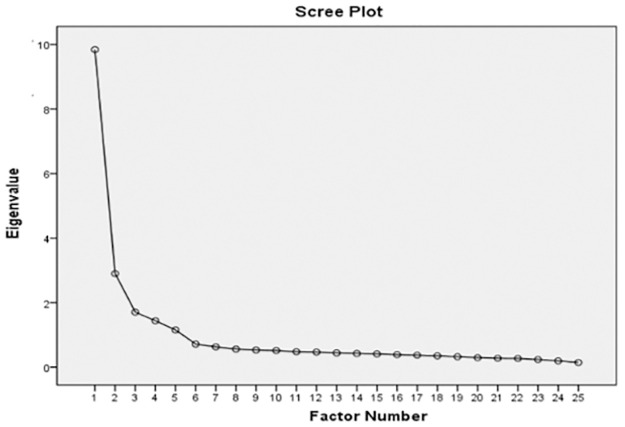
Scree plot result.

The third step is to determine the number of factors through two criteria: eigenvalue and scree plot. The combination of these two criteria helps with the final decision on the number of factors. The result shows that five factors are derived as their eigenvalues are greater than 1.00 [34, 39], and these five factors cumulatively account for 60.342% of the total variance of all 25 indicators, which is greater than the rule of thumb of 50% [40]. Furthermore, Fig 3 presents the scree plot result. As the sharp break occurs after the sixth factor, it confirms the five-factor solution. Therefore, both results suggest that five factors should be determined.

The fourth step is to rotate factors to find out data structures. As factor structures are related, the Oblique rotation was utilized, and the Direct Oblimin approach in the SPSS was employed [41]. The result also demonstrates the five-factor structure matrix. Items B1 to B5 are from F2, items B6 to B10 are from F1, items B11 to B15 are from F5, items B16 to B20 are from F4, and items B21 to B25 are from F3. All factor loadings are above .50 [34], which is acceptable.

The last step is to interpret, and all factors need to be labeled. [42] claims the determination of factor labeling should conform to “the principles of simplicity and interpretability” (p. 43). Also, the labeling factors could be the previously selected factors [43, 44] that are based on the theoretical model or framework. As such, the five factors are still named as perceived validity (F2: B1 to B5), absence of bias (F1: B6 to B10), access (F5: B11 to B15), administration (F4: B16 to B20), and social consequences (F3: B21 to B25) in the current research.

#### 4.2.2 CFA results

**Fig 4:**
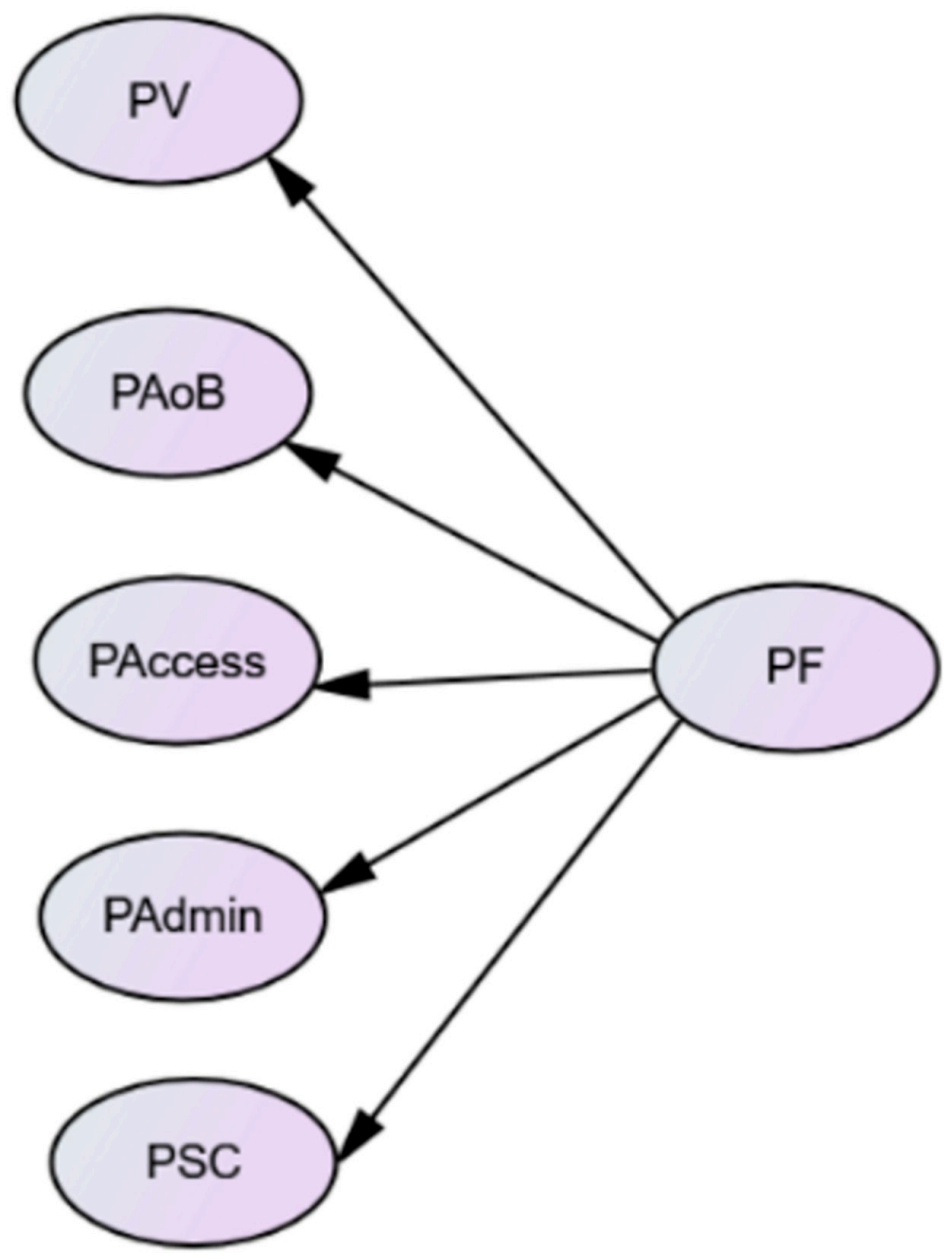
Hypothesized measurement model. Note. PF = Perceived fairness; PV = Perceived validity; PAoB = Perceived Absence of bias; PAccess = Perceived access; PAdmin = Perceived administration; PSC = Perceived social consequences.

CFA was, then, examined at the sub-scale level to assess the reliability and internal validity of each measurement model. The rest 675 participants’ responses were used. The purpose of CFA is to investigate whether the factors generated from EFA have the same underlying structure as the intended measurement structure. The perceived DET fairness is hypothesized as a model with one second-order latent factor, and five first-order latent factors. The second-order factor represents overall perceived fairness, and the five first-order latent factors with 25 items stand for perceived validity, perceived absence of bias, perceived access, perceived administration, and perceived social consequences. Each observed variable is hypothesized to load on only one latent factor (see Fig 4).

There are four steps to conduct the CFA [45]. The first step is to check the normality. Both univariate normality and multivariate normality are calculated before further analysis. The IBM^®^ SPSS^®^ AMOS v.26.0 is adopted to calculate the multivariate normality, and the Mardia’s test is used. The result of multivariate normality measurement shows the kurtosis is 329.862, which is far beyond the cut-off value -10 to 10 [46, 47]. Therefore, the normal distribution is violated, and the bootstrapping method [48] is subsequently performed in the following analysis.

The second step is to draw the measurement model (also see Fig 4), and the third step is to request the output from the software and report the CMIN/DF, RMSEA, CFI, and SRMR [49]. These steps were conducted through AMOS v.26.0. The last step is the interpretation. It is vital to report whether the latent variables are reflections of the associated observed variables. That said, it could be concluded whether the model has a good fit. As summarized in Table 3, model fit indices suggest the hypothesized model fits the data reasonably well (CMIN/DF = 1.961, RMSEA = .044, CFI = .901, SRMR = .032). To be noted, the standardized regression weights of B5 (β = .38), B10 (β = .57***), B17 (β = .67***), B19 (β = .61), and B24 (β = .48) are lower than the preferrable value .70 [38], and the residuals of B5, B19, and B24 are not significant. These three items are, therefore, eliminated for further SEM analysis.

**Table 3:** Model fit indices.

Model	CMIN/DF	RMSEA	CFI	SRMR
Fig 4	1.961	.044	.901	.032
Rule of thumb	< 3 Carmines and McIver [50]	< .08 Moss et al. [51]	> .90 UCLA Statistical Consulting [52]	< .08 Marquier [53]
Acceptability	Acceptable	Acceptable	Acceptable	Acceptable

Note. CMIN/DF = Minimum discrepancy per degree of freedom; RMSEA = Root mean squared error approximation; CFI = Comparative fit index; SRMR = Standardized root mean squared residual

#### 4.2.3 SEM results

**Fig 5:**
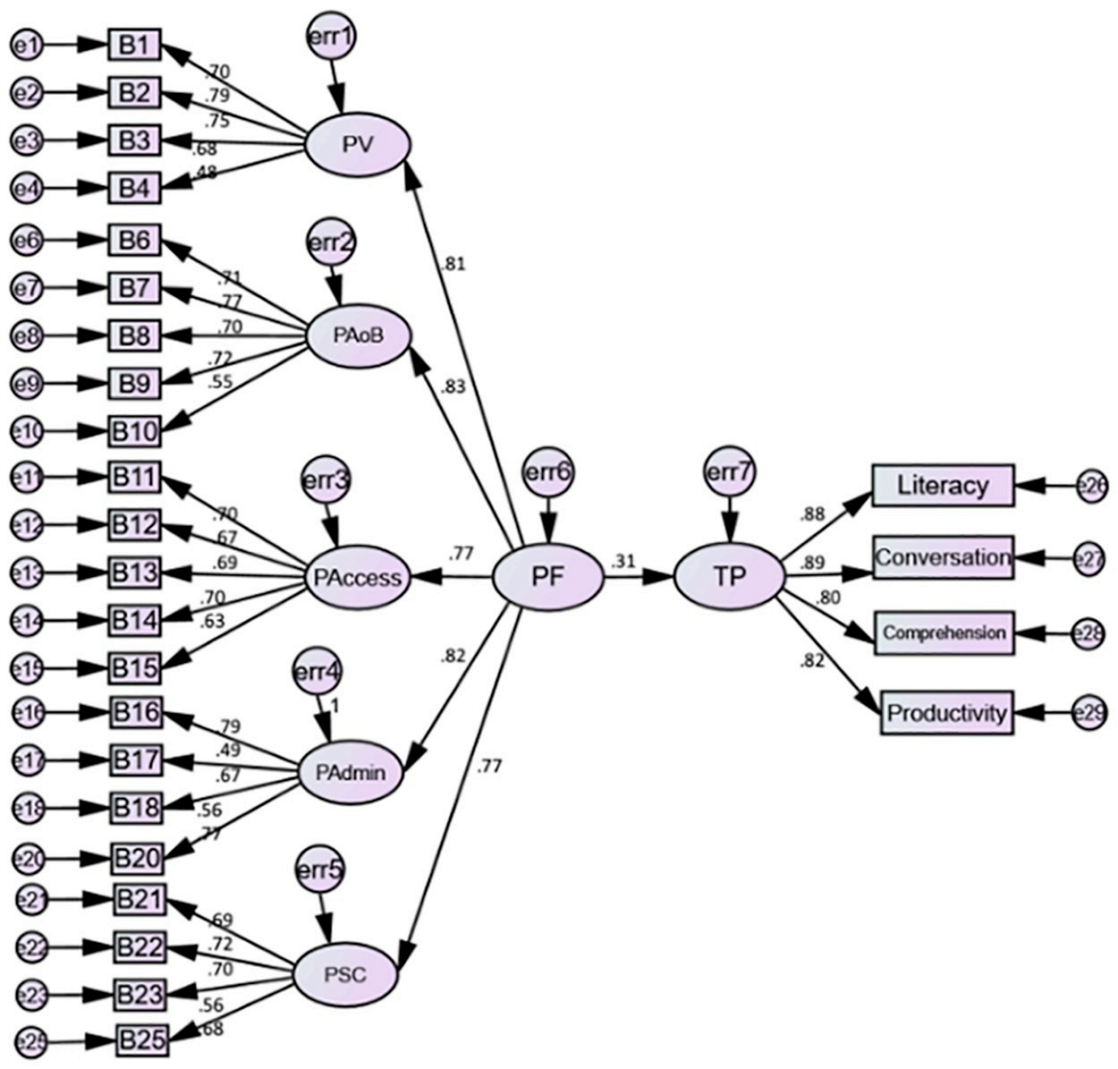
Final structural equation model with standardized parameters. Note. PF = Perceived fairness; PV = Perceived validity; PAoB = Perceived Absence of bias; PAccess = Perceived access; PAdmin = Perceived administration; PSC = Perceived social consequences; TP = Test performance.

SEM was, finally, applied to model the hypothetical relationships between perceived DET fairness and test performance. The result turns out a reasonable model fit (CMIN/DF = 2.159, RMSEA = .052, CFI = .891, SRMR = .045). Though, the CFI value is a little lower than the preferable value, as it is quite close to .90 and considering the complexity of the model, it is deemed admissible. Fig 5 displays the final structural equation model with standardized regression weights.

Also, as illustrated in Table 4, test takers’ perceptions of DET fairness have a significantly positive association with perceived validity (β = .808, p< .001), perceived absence of bias (β = .831, p< .001), perceived access (β = .773, p< .001), perceived administration (β = .819, p< .001), and perceived social consequences (β = .770, p< .001). Besides, test takers’ perceptions of DET fairness have a positive effect on test performance (β = .312, p< .001), denoting that test takers who perceived the DET as fairer performed better in the test.

**Table 4:** Standardized path coefficients results.

Path(s)	SPC (β)	S.E.	C.R.	p
PV <--- PF	.808	.084	9.178	***
PAoB <--- PF	.831	.068	12.088	***
PAccess <--- PF	.773	.066	11.767	***
PAdimin <--- PF	.819	.071	11.456	***
PSS <--- PF	.770	.084	9.332	***
TP <--- PF	.312	.057	5.824	***

Note.

*** represents p< .001;

** represents p< .01;

* represents p< .05;

SPC = Standardized path coefficients (β); S.E. = Standardized error; C.R. = Critical ratios

## 5 Discussion

- **RQ1: How do test takers perceive the fairness (i.e.**, **validity, absence of bias, access, administration, and social consequences) of the DET?**

RQ1 is concerned with test takers’ perceptions of DET fairness, and [27]’s TFF comprising perceived validity, perceived absence of bias, perceived assess, perceived administration, and perceived consequences is used as the theoretical basis for discussion. In the previous section, it was found that test takers perceived the DET to be fair overall, and they perceived that they had equal access to the test. This result is not surprising because DET, as one of the first at-home tests, has been intensively validated by researchers and scientists from its company. Besides, test takers perceived that the price of the DET was appropriate. This result is also understandable as the DET costs 49 U.S. dollars, and the test fee is much more affordable to test takers compared to other standardized proficiency tests such as IELTS and TOEFL (around 300 U.S dollars in China). Furthermore, test takers perceived that choosing test time themselves made taking the test easy. For one thing, the time-of-day of taking the test is omitted in Kunnan’s [27] TFF, but it is stressed by other researchers such as Ammons and Booker [54] and Hines [55]. They both concluded in their research that time-of-day played an important role in students’ achievements. Specifically, students scored higher on the test if they chose the test time themselves. Given that test takers in the current study also perceived the importance of time-of-day, this variable could not be ignored in the TFF or in the fairness research; for another, choosing the test time by test takers allows more flexibility and curtails possible problems of fairness so that they could better prepare for the test, and test takers usually know the time that could perform better [54]. For this reason, the current study argues that the variable time-of-day should be added to the TFF.

For the absence of bias quality, test takers perceived that items on the DET were not biased in terms of gender and religious belief differences. This result resonates with Maris’ [8] DET Research Report outcomes with the conclusion that the DET items are not biased toward different groups of test takers. Besides, based on the test fairness definition in the Standards for Fairness and Quality [56], free of bias of the DET was achieved as construct-irrelevant personal characteristics were minimized and had no negative effect on test takers’ performance. Hence, it may arrive at the conclusion that the DET is both objectively fair (showed in the previous studies) and subjectively fair (demonstrated in the current study) in terms of test items as they do not favor any group of test takers. For the administration quality, test takers perceived that the DET provides different forms to test takers. Using different test forms may alleviate the possibility of cheating on the test [5, 17] because different test takers would receive different tasks or items. By doing so, the test security could be improved. It is argued by DET developers that the DET is a computer-adaptive test, so different test takers have different test items based on their performance. Also, they also claim that all test items are extensively validated and different test forms at the same level are parallel. For the social consequences quality, test takers perceived that their English proficiency was improved after taking the DET, and the DET helped their English learning. Wagner [4] contends there is no research that has mentioned the washback or consequences of the DET. This is an important omission, though. Washback, broadly defined as the effect of an assessment on classroom-based teaching and learning [57, 58], has been researched extensively in the field of language assessment over the past few decades. Additionally, it is believed that high-stakes and large-scale assessments are more likely to exert intensive washback, and it is more valuable to probe the nature of washback in terms of these kinds of tests [59, 60]. As the DET has both qualities, it is necessary to investigate its washback to signal a more understanding of how the DET influences teaching and learning. Therefore, it is hoped that further research could lay more emphasis on the washback of the DET.

However, test takers perceived that the DET was not valid. Specifically, test takers perceived that the content on the DET may not be appropriate to measure their language ability. This result is in line with [4]’s view that the DET has little in common with the university contexts though it is widely used for university admission purposes, and it is also consistent with Isbell and Kremmel’s [5] viewpoint that DET is not associated with real-life demands. The result in the current study, therefore, implies that DET developers may need to focus more on real-life situations to fit test takers’ use in the target language use (TLU) domain to ensure the content on the test is appropriate for test takers. Besides, test takers perceived that their DET scores could not be aligned to IELTS or TOEFL scores. This result is contradicted with the outcomes from DET Research Reports as most of which concluded that the DET scores are moderately to largely correlated with IELTS or TOEFL scores (e.g., [14, 15]). Isbell and Kremmel [5] argue that, though, the DET score is moderately to largely correlated to the IELTS or TOEFL score, the interchangeable use of scores needs further negotiation. Also, one participant in the interview, who attended both DET and IELTS, believed that the score she obtained in the IELTS was more reliable than that in the DET. She further explained it might be because of test duration (DET is around one hour and IELTS is around three hours). Her interpretation was that a longer duration of the test may better examine comprehensive language abilities. This leaves an important implication for researchers to investigate whether a longer test duration could better reflect test takers’ real language abilities, or whether test duration would influence test takers’ test performance.

- **RQ 2: How does perceived DET fairness affect test performance?**

The result showed that test takers’ perceptions of DET fairness significantly affected their test performance, suggesting that test takers who believed the DET as fair performed better on the test. To be more specific, perceived validity positively affected test performance. Xie [23] contends that practicing valid test papers could be a logical way to enhance test performance and language ability. However, test takers perceived that the DET was not valid in terms of its content and criterion in the current study, which leaves a vital implication for DET developers to lay more emphasis on the perceived validity of the DET as it influenced test preparation. Previous DET Research Reports (e.g., [14, 15]) have demonstrated that the DET scores could be aligned to IELTS or TOEFL scores, but they should strive hard to investigate stakeholders’ views of DET validity. Because stakeholders are affected by an assessment the most, their views also attach great importance [11, 12]. In other words, it is suggested that DET experts may not only research the objective perspective (i.e., psychometric examinations of the test) of the DET validity, but also investigate the subjective perceptive (i.e., stakeholders’ perceptions) of the DET validity.

Additionally, perceived access positively affected test performance. This result is understandable as, nowadays, students have various resources to access the test. For instance, educationally, students can download practice DET test papers from the official website, and many websites (e.g., Dengdeng Duolingo) offer both real and mock test papers; financially, the DET costs 49 U.S. dollars. Compared with the cost of other standardized tests such as IELTS and TOEFL, the DET is more affordable to most test takers; geographically, students could take the DET anywhere as the test is at-home. However, for those in-person tests, if there is no test site in the students’ city, they need to go to another city to take the test. That said, they might cost extra time, energy, and money, and might not be able to perform the best due to the influence of these extra issues; as for conditions and equipment, students are allowed to use their own personal devices to take the test, and they may not need to worry about the familiarity to the device. Again, though IELTS and TOEFL also provide the online mode, test takers are required to use the computer in the test room.

Moreover, test takers’ perceptions of absence of bias positively affected test performance, meaning that test takers who endorsed more in absence of bias tended to outperform in the test. In the current study, test takers perceived that DET items were not gender- and religion-biased. As such, they may perform better because they found that test items were equal to everyone. Then, test takers’ perceptions of administration positively affected test performance. Test administration of the DET is always an important part as the DET is an entirely at-home test. Isbell and Kremmel [5] claim that the test security of the DET is rather rigorous, and the test has high technological demands to ensure its security, e.g., eyes focus on the camera, headphone and mobile phone prohibition, and browser exit prohibition are all the requirements of the invigilation. Also, students may need to consider the physical conditions of the room such as light and temperature. These all suggest that test takers who perceived the importance of test administration tended to engage more in the test for a better performance. Finally, test takers’ perceptions of social consequences positively affected test performance. The occurrence of this phenomenon may be because most test takers took the DET for university admissions purposes. If they knew the test would be helpful for their English learning, they might strive harder to get a higher score in the test.

## 6 Conclusion

The current study examined the perceived DET fairness and its effect on test performance and found that test takers perceived the DET to be fair overall, and they perceived that they had equal access to the test, but the test was not valid. Besides, perceived DET fairness had a positive effect on test performance with the perceived validity and perceived access having the strongest effects. Hopefully, it brings about the following implications to the field: (1) theoretically, it is argued that the variable time-of-day should be added in the TFF; (2) methodologically, a DET Fairness Questionnaire focusing on subjective test fairness was developed which could be used for further test fairness research; and (3) practically, major stakeholders could be benefited, e.g., test developers could design fairer and more authentic test papers to provide a better testing environment for test takers. Nevertheless, several limitations are worth noting and the agenda for further research is suggested. To begin with, the demographic information of participants was diverse, but the study did not conduct a multi-level analysis of the data. Therefore, further research is suggested to consider more demographic information (e.g., gender, region, and English proficiency level) to obtain a comprehensive understanding of the perceived test fairness and its effect on test performance. Additionally, the study used a questionnaire as the principal instrument, which means the data gleaned were primarily self-reported data. Self-reports without any in-depth understanding from participants may cause reliability problems; and statistical analyses of these self-reported data are usually quantitative, but the relationships among different theoretical concepts are not necessarily quantitative. For this reason, further research is recommended to conduct follow-up interviews to access participants’ deep thoughts based on their questionnaire responses. To conclude, it is to be hoped that researchers could bolster studies on subjective test fairness as it is a promising research area in language assessment.

## Supporting information

S1 Appendix (PDF)
