# Supplementary material for: Examining the subjective fairness of at-home and online tests: Taking Duolingo English Test as an example
Source: PLoS One. 2023 Sep 19;18(9):e0291629. doi: 10.1371/journal.pone.0291629 (PMC10508603; doi:10.1371/journal.pone.0291629)
Supplement: S1 Appendix — (PDF) [file pone.0291629.s001.pdf]

APPENDIX
DET Questionnaire (English version)

- **Section A: Demographic Information**

**Gender**	**Age**
**Region**	**First language**
**Second language**	**Years of learning English**
**Times of taking DET**	**Latest DET date**
**Latest DET scores**	Total: __________<br>Sub-scores:<br>Literacy ___________ Comprehension _____<br>Conversation _______ Production ____

- **Section B: Perceptions of DET Fairness**

This section aims at understanding your perceptions of DET fairness. Please read each statement and choose one (1, 2, 3, 4, 5, or 6) that tells HOW MUCH YOU AGREE WITH THE STATEMENT.

1 = Strongly disagree, 2 = Disagree, 3 = Slightly disagree, 4 = Slightly agree, 5 = Agree, and 6 = Strongly agree

e.g., Learning English is very important. 1 2 3 4 5 (6)

**No.**	**Content**	**1**	**2**	**3**	**4**	**5**	**6**
B1	The DET is an adequate measure of my language ability.	1	2	3	4	5	6
B2	The content on the DET is appropriate to measure my language ability.	1	2	3	4	5	6
B3	The DET score is comparable to other proficiency tests such as IELTS and TOEFL.	1	2	3	4	5	6
B4	My score is appropriate for the performance I have on the DET.	1	2	3	4	5	6
B5	Automated scoring of the DET is reliable.	1	2	3	4	5	6
B6	Pronunciation of the speakers on the test did not help some test takers or hurt others.	1	2	3	4	5	6
B7	Test items or tasks did not favor male or female test takers.	1	2	3	4	5	6
B8	Test items or tasks did not favor test takers with different religious beliefs.	1	2	3	4	5	6
B9	Test items or tasks did not favor younger or older test takers.	1	2	3	4	5	6
B10	The criterion setting of the DET score does not favor anyone.	1	2	3	4	5	6
B11	The Duolingo, Inc. provides sufficient test preparation materials to test takers.	1	2	3	4	5	6
B12	The price of the DET is appropriate.	1	2	3	4	5	6
B13	Choosing the test location myself made it easy to take the test.	1	2	3	4	5	6
B14	Using my own laptop made it easy to take the test.	1	2	3	4	5	6
B15	Choosing the test time myself made it easy to take the test.	1	2	3	4	5	6
B16	Controlling the physical conditions under which I took the test made sure I gave my best performance.	1	2	3	4	5	6
B17	Test takers should take different forms of the test.	1	2	3	4	5	6
B18	The Duolingo, Inc. provides accommodations for disabled test takers.	1	2	3	4	5	6
B19	The procedures of the test (steps to complete the test) are equal for all test takers.	1	2	3	4	5	6
B20	It is possible to cheat in the exam.	1	2	3	4	5	6
B21	I am able to *appeal my score if I want to.	1	2	3	4	5	6

	(*Appeal means if you find your score is wrong, you are able to ask the company to re-score and re-evaluate the test responses.)						
B22	My English proficiency can be improved by the DET.	1	2	3	4	5	6
B23	The DET helps my English learning.	1	2	3	4	5	6
B24	The DET enhanced my test-taking strategies.	1	2	3	4	5	6
B25	I acquired more learning methods with the help of DET.	1	2	3	4	5	6
